# Training Load, Mileage, and Perceived Exertion as a Predictive Model of Injury and Illness in Women’s Soccer

**DOI:** 10.3390/sports13110411

**Published:** 2025-11-17

**Authors:** Corbit Franks, Andrew Yockey, Nicholas Bosley, Tyler Myers, Kaitlyn Armstrong, Melinda Valliant, Chip Wade

**Affiliations:** 1 Human Movement & High-Performance Lab, The University of Mississippi, Oxford, MS 38677, USA; nbosley@olemiss.edu (N.B.); tmmyers1@go.olemiss.edu (T.M.); karmstr3@olemiss.edu (K.A.); cwade@olemiss.edu (C.W.); 2 Department of Health, Exercise Science, and Recreation Management, The University of Mississippi, Oxford, MS 38677, USA; 3 Center for Health and Sports Performance, The University of Mississippi, Oxford, MS 38677, USA; valliant@olemiss.edu; 4 Department of Public Health, The University of Mississippi, Oxford, MS 38677, USA; rayocke1@olemiss.edu; 5 Department of Nutrition & Hospitality Management, The University of Mississippi, Oxford, MS 38677, USA

**Keywords:** training load, exertion, injury, illness, soccer

## Abstract

This study examined the relationship between training load, mileage, and session rating of perceived exertion (s-RPE) as predictors of injury and illness in Division I women’s soccer players. Twenty-four athletes were monitored over a 13-week season including 69 athlete exposures (49 training sessions and 20 matches). Internal and external load were measured during each athlete exposure. Player injury and illness status were documented daily by medical staff and categorized as healthy, medical attention, or time-loss. Associations between athlete exposures and injury/illness status were analyzed using a mixed-effects ordinal logistic regression model with player ID as a random intercept. A total of 1560 athlete observations were included. Higher daily mileage was associated with increased odds of injury or illness (OR = 1.67, 95% CI: 1.19–2.34). Training load was associated with reduced odds of injury or illness, with each unit increase lowering the odds by 42% (OR = 0.58, 95% CI: 0.41–0.83). Session-RPE was not significantly associated with injury or illness (OR = 0.96, 95% CI: 0.65–1.42). These findings indicate that accumulated mileage elevates injury and illness risk, while structured increases in training load enhance athlete resilience, and reduce injury and illness risk. Monitoring both internal and external workload provides performance staff with a practical approach to optimize training stress, augment recovery, and prepare athletes for the demands of competition in women’s soccer.

## 1. Introduction

Participation in women’s sports has grown substantially, with women’s soccer continually growing in the number of teams and players at the National Collegiate Athletic Association (NCAA) level. According to the NCAA research database, participation in women’s athletics across all divisions has increased by over 28,000 individuals in the last 10 years with a 12% increase in Division I and setting a record number of athletes during the 2023–2024 year [1]. As participation in women’s sports increases, injuries increase as well. Women’s soccer is the leading sport in injury rates across female athletics [2]. The NCAA Student-Athlete Health and Wellness Study (2023) reported that 54% of female soccer players across all divisions sustained at least one major injury, defined as a sport-related injury that required surgery, hospitalization, or more than one month away from training or competition [3]. NCAA women’s soccer had an injury rate of 8.65 injuries per 1000 athlete exposures (AEs) between practices and competitions [4]. Additionally, 39% of all injuries resulted in time loss of greater than one day. This uneven increase exposes an issue in training programming that puts these athletes at a greater risk of injuries.

In recent years, female athletes have come to represent a much larger portion of the athletic community; however, research efforts on female athletes have not advanced at the same rate. A scoping review highlighted the disparity of research on female athletes when compared to their male counterparts reporting 16% of the literature including female populations [5]. Few studies have highlighted sex-specific differences in performance, workload, work capacity, or injury and illness rates between male and female athletes [6,7]. While some aspects of the current literature may be applicable across sexes, the aforementioned sex-specific differences in performance must be accounted for in research and subsequent training programs. By tailoring research initiatives to the unique biological and physiological characteristics of female athletes, clinicians aim to support global efforts to reduce gender disparities in sports and enhance the overall quality of care and training for this population. Previous research suggests that acknowledging and addressing these disparities can improve health and performance outcomes while reducing injury rates among female athletes [8,9].

Differences in training loads between male and female soccer players during practices and games have also been reported. During competition, both groups had similar relative workloads; however, men covered greater distances at higher velocity thresholds (M: 21.9 ± 3.2 sprints, F: 13.8 ± 5.0 sprints; *p* < 0.05), which created a different physiological load, while women covered more total distance at lower velocity (M: 2116.4 ± 508.3 m, F: 2584.5 ± 311.1 m; *p* < 0.05) [10]. This contrast in playing style illustrates the difference in accumulated workload and supports the need for sex-specific approaches to training and recovery.

Monitoring an athletes’ workload has become a popular practice across all levels of sport and physical activity. Athlete workloads have been shown to have a relationship with sport specific performance in both individual and team outcomes [11]. Workload can be described as external or internal. External workload comprises the physical work that an individual performs including speed, power output, velocity, or distance covered. Internal workload encompasses the physiological and psychological response to external stimuli such as self-perceived effort or session-reported rating of perceived exertion (s-RPE), heart rate (HR), and hormonal changes [12]. Workload measurements can provide practitioners with tangible data that may be translated directly to the field through the individualization and optimization of training programs and reduction in injuries [13,14,15]. Athlete workload assessments are multifactorial in nature and should combine all sport-specific on-field training, strength training, and recovery to assess the true work capacity of an athlete across a single session, day, or extended period. Quantifying total workload can be a difficult practice and many times fails to consider outside-of-sport activities. Workload capacity and total workload calculations may also fail to account for an athlete’s recovery and regeneration techniques such as total caloric and macronutrient intake, hydration, sleep duration and quality, and perception of and tolerance for training [16,17,18,19].

Several studies have suggested the use of s-RPE to quantify internal load and provide an accurate measure of athlete workload [20,21,22,23]. One study further evaluated the use of s-RPE as an internal measure of workload by having American football athletes evaluate their s-RPE immediately following the day’s session and by performing a reactive strength index test (RSI) the following day [19]. RSI scores were then correlated with s-RPE as an attempt to measure recovery. A significant association was shown between higher reported s-RPE and lower RSI values, indicating that s-RPE is an accurate depicter of workload and residual needs for recovery. Similarly, s-RPE was shown to correlate to total number of sprints, distance covered, and mechanical load [24]. This correlation describes a relationship between internal and external loads.

The use of Global Positioning System (GPS) measurement for external load has been validated through a systematic review that included 38 studies and found significant evidence for associations between training load and injury risk [25]. Acute: chronic workload ratios (ACWRs) were examined across four weeks of accumulated training using GPS-derived measures to quantify external load and its relationship with injury risk. They found that high one-week values for total distance and workload were associated with increased injury risk, whereas high absolute values with a low ACWR were associated with decreased injury risk [20]. The findings highlight the importance of progressing weekly loads at an appropriate rate—keeping weekly acute exposure low while building chronic tolerance. Additionally, another study reported that high ACWRs (>2.0) were associated with increased injury risk, while moderate ACWRs were associated with reduced risk [22]. This association aligns with a systematic review that describes a U-shaped curve, indicating that ACWR are either significantly higher or lower than moderate values (1.00–1.25) are associated with increased injury risk [26]. In contrast, Impellizzeri et al. (2021) [27] argued that the ACWR is merely a rescaling of the acute load and does not accurately reflect changes in injury risk. Using randomly generated chronic workload values with previously collected ACWR data, they demonstrated that the same associations persisted, suggesting the ratio’s lack of true predictive power [27]. This disparity in the reliability and validity of the ACWR underscores the need for more robust and theoretically sound approaches to predicting injury risk.

The consistent underrepresentation of female athletes in sports science research has limited the development of sex-specific strategies for injury reduction and workload management. As injury rates in women’s soccer continue to rise, it is essential to identify factors that can accurately predict injury and illness risk within this population. This study aims to address this by investigating the predictive relationship between internal and external workload measures and the incidence of injury and illness in female collegiate soccer players throughout a competitive season. Findings from this research may contribute to the development of more individualized training and recovery strategies that enhance performance while reducing injury risk among female athletes.

## 2. Materials and Methods

A longitudinal design was used to collect data from 24 female collegiate soccer players over 13 weeks, which resulted in 49 practices and 20 matches. Participants were monitored using a Polar Team Pro GPS heart rate monitor. Data collected included mileage, training load, and s-RPE. Team medical staff recorded all medical encounters and assigned values for health status: healthy, injury/illness, or time-loss injury/illness. The relationship between training exposure and risk of injury/illness was analyzed using a mixed-effects ordinal logistic regression model. The dependent variables were coded as healthy = 0, injury/illness = 1, and time-loss = 2. Independent variables included mileage, training load, and s-RPE.

Twenty-four sub-elite amateur female collegiate soccer players (age range, 18–22 years) from a National Collegiate Athletic Association (NCAA) Division I power 4 school participated in this study. All participants met the inclusion criteria of being deemed healthy, injury free, and physically fit according to a pre-participation physical exam (PPE) and cleared for full participation in sport activities by licensed medical professionals. This study was approved by the University Institutional Review Board and pursuant to policy 45 CFR 46.117 (c)(2), and the requirement for signed informed consent was waived; however, verbal informed consent was received to utilize data for research purposes.

Data was collected over the course of one competition season to include pre- and regular season training sessions and competitions. Researchers observed a total of 1560 participant encounters spanning 13 weeks and 69 days of training and competition. Participants averaged 3.5 training sessions and 2 competitions per week during this time period. Prior to the onset of data collection, all participants underwent a PPE (pre-participation exam) to ensure they met study inclusion criteria. Following inclusion in the study, each participant was educated in the use and parameters of the Polar Team Pro system (Polar Electro Oy, Kempele, Finland, 1977), the Polar Team Pro GPS heart rate monitor sensor, and the self-reported rate of perceived exertion scale.

Each participant was assigned a Polar Team Pro profile and sensor then properly fitted for the accompanying Polar Team Pro sensor chest strap. PPE measures such as height and weight were input in their assigned Polar Team Pro profile to aid in precise data calculations. Participants were then instructed to wear their assigned sensor to all individual and team training sessions as well as team competitions. Compliance with this measure was confirmed by team performance staff through Bluetooth connectivity of each subject’s sensors and live data recording and presentation in the Polar Team Pro application on an Apple iPad. Sensors collected a variety of data points to include distance (in meters and converted to yards/miles), average heart rate, maximum heart rate, session duration, time in heart rate zones, average speed, maximum speed, time in speed zones, accelerations and decelerations, and an algorithm calculated training load for each session. According to Polar Electro (Polar Electro Oy, Kempele, Finland, 1977), the training load algorithm was designed to quantify the difficulty of each session and is based upon the subjects’ internal and external load experienced during the session. The combined internal and external loads include overall and average heart rate and heart rate zones, predicted caloric expenditure, mechanical impact (high-speed accelerations and high-speed decelerations) and duration of the activity to calculate a single understandable external load output [28]. The Polar Training load calculation is further based on the Banister training impulse model or TRIMP model and provides a numerical value for the athletes’ response to a single training session. Sensors were collected after all activity and data was extracted and synced with the Polar Team Pro dashboard and each subject’s assigned profile. Training load scores and mileage were exported into a data collection spreadsheet in Microsoft Excel.

**Figure A1:**
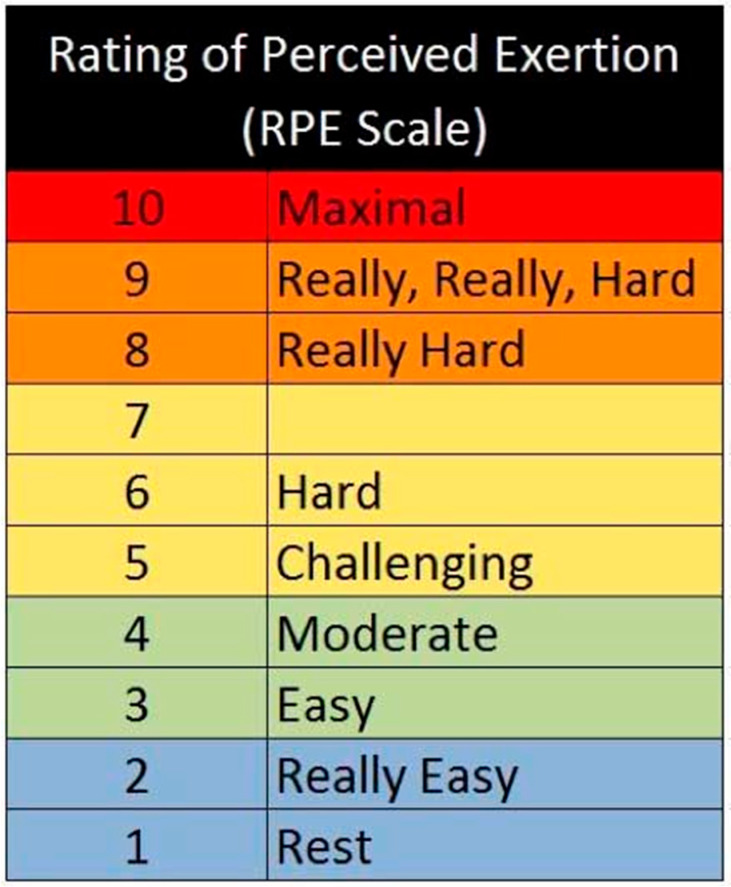
s-RPE Scale.

Performance staff members collected each participant’s s-RPE after each training session and competition as a measure of perceived internal load. The use of the s-RPE has been well researched and shown to be an accurate measure of internal load in women’s soccer [29]. Athletes were shown a color-coded s-RPE scale and asked to rate their individual perceived difficulty of the session or game (Appendix A, Figure A1). The s-RPE scale included ratings of 1–10 and included descriptors such as rest 1, easy 3, moderate 4, challenging 5, hard 6, really hard 8, and maximal 10. The color coding went from blue at s-RPE 1 to green at s-RPE 3, yellow at s-RPE 5, orange at s-RPE 8, and red at s-RPE 10. Athletes were instructed to assess their own value for each session and not consult teammates regarding their s-RPE score to eliminate potential inaccuracies or bias of the determined rating. Scores were collected approximately 10 min after the end of training or competition so that the volume, intensity, and duration was still fresh in each participant’s mind, and there was no buffering effect from the team cooldown on the session ratings. The s-RPE scores were recorded in the data collection spreadsheet.

Injuries and illnesses were documented by the team medical staff and quantified as a medical encounter with the athletic trainer in which the athlete received treatment and was monitored during any team training activity or competition. Furthermore, injuries and illnesses were categorized based on severity and whether the episode required removal from training sessions or competition. All injuries and illnesses were documented in the participants’ electronic medical record and extracted into an excel spreadsheet.

The outcome variable was daily injury/illness status, coded as an ordinal variable with three categories: 0 = healthy, 1 = medical attention injury or illness (MAI), and 2 = time-loss injury or illness (TLI). Primary predictors included daily mileage, training load (derived from GPS and heart rate data), and s-RPE.

To examine the relationship between training exposures and injury or illness risk, we used a mixed-effects ordinal logistic regression model. Previous work by Akubat, Barrett, and Abt (2014) [30] suggested that integrated ratios of internal and external load may be more informative than assessing either component in isolation. This supports our use of combined internal and external metrics to develop a predictive model of injury and illness risk [30].

A random intercept for player ID was included to account for within-subject correlation over time. Analyses were restricted to complete cases, assuming missing data were missing at random [31,32]. Results were reported as odds ratios with 95% confidence intervals. Intervals were considered statistically significant if they excluded the null value (OR = 1.0) [33]. Model assumptions were assessed and were not violated. Statistical analyses were conducted using R Studio 2025.05.0 using the “brant”, “MASS”, “ordinal”, and “lme4” package.

## 3. Results

A total of 1560 observations were included in the analysis. The team means for mixed, external, and internal load across the 1560 observations were as follows: training load 122.10, distance 2.60 miles, and s-RPE 3.96. Detailed means for each participant can be found in Table 1, and averages across the microcycles can be found in (Appendix A, Table A1). Results from the mixed-effects ordinal logistic regression model shown in Table 2 indicated that higher daily mileage was associated with increased odds of more severe injury or illness. Specifically, each unit increase in mileage was associated with 67% higher odds of progressing to a more severe injury/illness status (OR = 1.67, 95% CI: 1.19–2.34). This suggests a dose–response relationship between mileage and injury severity.

In contrast, higher training load was associated with decreased odds of injury or illness severity. For each unit increase in training load, the odds of reporting a more severe injury or illness decreased by 42% (OR = 0.58, 95% CI: 0.41–0.83). Session rate of perceived exertion (s-RPE) was not significantly associated with injury/illness severity (OR = 0.96, 95% CI: 0.65–1.42).

## 4. Discussion

Higher training load was associated with a protective effect, significantly decreasing the odds of more severe injury or illness by 42% per unit increase (OR = 0.58, 95% CI: 0.41–0.83). This finding may seem counterintuitive at first, but it reflects emerging perspectives on training load management. This may suggest that athletes with structured increases in training load, possibly representing better conditioning or periodization, tend to demonstrate greater resilience and lower injury severity [23,34]. Athletes who progressively increase training load within adaptive capacities may develop physiological robustness that buffers against severe injury or illness [35]. This finding also echoes the concept of the “training-injury prevention paradox,” where appropriate training loads decrease injury risk, whereas abrupt spikes or chronic undertraining can predispose athletes to injury [34]. Additionally, athletes exposed to higher training loads are more likely to develop physical qualities associated with a reduced risk of injury, including increased strength, high intensity running capacity, and improved cardiovascular fitness. This evidence suggests that progressive exposure to higher training loads enhances the body’s ability to tolerate such demands [36].

The results of this study align with previous research, concluding that increased distance correlates with increased risk of injury. This increased risk of injury has been shown across multiple sports. For example, distance runners who increase session-specific distance greater than 10% of their longest run from the previous 30 days significantly increase the risk of lower extremity injury [37]. Other studies assessed distance over time as a predictor of increased injury risk rather than total distance [38,39]. Future studies should assess the relationship between overall distance and the accumulation of high-speed distance as they relate to injury risk. Furthermore, performance specialists should focus on gradual increases in both total distance as well as high-speed running distance, while ensuring that athletes are subjected to match speed during pre-season preparation and return to play.

Session rate of perceived exertion has been shown to be a valid tool to measure the workload or difficulty of a single training session [21,40]. While data analyzed in this study show no significant association between the s-RPE and injury or illness severity, researchers do not believe the lack of association negates validity of s-RPE as a tool to measure workload. However, there may be caveats to s-RPE that may still need to be addressed in this population. A review of the current literature shows that while the s-RPE has been well studied, there is limited data on female athletes [40]. Female athletes remain significantly underrepresented in the literature at large and this gap limits both the understanding and the efficacy of validated tools to this population and effects the limited reproducibility of much of the peer-reviewed literature available in sport and exercise [5,9]. Furthermore, the accuracy of s-RPE may be related to altered mood states, outside stressors, acute spikes in training demands, chronotypes, or the phases of the menstrual cycle [40,41,42]. In collegiate athletes, there is also the uncertainty of coaching perception, and peer-pressure that may further inflate or deflate the s-RPE scores, creating inaccurate reporting [43].

Future research should aim to harness artificial intelligence and machine learning to formulate athlete and sport specific readiness scores and recovery protocols based on internal and external load measures. Research continues to show that time-loss creates disparity across sport [44]. Time-loss injury and illness may be attributed to a variety of factors including sleep quality and duration, perceived fatigue, academic and personal stress, and perceived mood [45]. As time-loss is a multi-faceted phenomenon, it will take a multi-faceted approach to mitigate the risk of injury and illness in sport [46]. While practitioners will likely never fully prevent injury and illness in athletes, the calculated use of internal and external load to assess athlete readiness and overall injury risk poses an opportunity to drive informed decisions, formulate practical recovery protocols, and reduce injury risk in sport. Furthermore, the results are intended to be predictive in nature and not to imply causation between workload metrics (e.g., RPE, mileage, and training load) and injury or illness in female athletes. Therefore, any observed links should be interpreted as associations that may be useful in the stratification of risk and not causation of injury and illness. The sample was a convenience cohort of athletes from a single university women’s soccer team, and an a priori sample size calculation was not conducted. The lack of calculation may reduce the generalizability to other teams, levels of play, or sports. Future studies should use prospectively powered, multi-team samples to validate and extend these findings.

## 5. Conclusions

Sport-related injuries and illnesses impair athletic performance, development, and team success. Performance staff must therefore design programs that reduce injury risk while enhancing performance. Findings from this study show that greater total mileage is associated with higher injury incidence, underscoring the importance of efficient workload management. Optimizing acute-to-chronic workload ratios, rather than relying on high training volumes, may reduce injury risk while promoting adaptation. High-intensity, low-duration sessions emphasizing neuromuscular activation can increase load capacity without compromising recovery during dense competition periods. Tailoring training to sport- and position-specific demands further improves readiness and resilience. Overall, these findings support prioritizing strategic intensity over volume through individualized, load-monitored programming to optimize performance and minimize time-loss injuries.

## Figures and Tables

**Table 1:** Participant and team mixed, internal and external load means.

Participant ID	Polar Team Pro Training Load	Mileage	s-RPE
1	66.7	2.08	6.01
2	147.52	2.22	3.98
3	88.35	1.96	2.8
4	163.46	3.65	3.91
5	164.57	2.44	4.48
6	84.62	1.76	2.34
7	146.93	3.16	4.33
8	127.79	2.96	3.9
9	158.7	2.58	4.3
10	128.97	2.55	4.64
11	114.35	3.44	4.19
12	59.45	1.91	3.35
13	109.61	3.26	4.16
14	82.72	1.96	2.97
15	121.99	2.48	4.87
17	136.62	2.3	3.97
18	112.57	2.76	4.28
19	188.19	3.47	3.88
20	127.29	1.6	2.75
21	61.48	1.71	2.81
22	71.38	2.35	3.67
23	157.3	2.96	4.6
24	196.19	4.09	4.22
25	107.51	2.61	4.32
Team Average	122.10	2.60	3.96

**Table 2:** Regression results.

Variable	OR	S.E.	95% CI
Mileage	1.67	0.17	[1.19, 2.34] *
Training Load	0.58	0.18	[0.41, 0.83] *
RPE	0.96	0.19	[0.65, 1.42]

* indicates *p* < 0.05.

## Data Availability

The data presented in this study are available on request from the corresponding author due to privacy restrictions.

## Abbreviations

The following abbreviations are used in this manuscript:

NCAA	National Collegiate Athletic Association
AEs	Athlete exposures
s-RPE	session-reported rating of perceived exertion
HR	Heart Rate
RSI	reactive strength index test
GPS	Global Positioning System
ACWR	Acute to Chronic Workload Ratio
PPE	pre-participation physical exam
MAI	medical attention injury or illness
TLI	time-loss injury or illness

## Appendix A

**Table A1:** Participant and team mixed, internal and external load means across Microcycles.

Participant ID	Microcycle	Polar Team Pro Training Load	Mileage	s-RPE
1	1	275	3.52	5.2
1	2	116.33	3.47	62.67
1	3	66.5	2.49	4.67
1	4	62.17	2.2	2.67
1	5	34.17	1.4	2.17
1	6	77.5	2.08	4
1	7	54.75	1.74	3
1	8	37.67	1.64	3.17
1	9	30	1.53	4
1	10	34.17	1.72	3
1	11	8.5	1.79	3.5
1	12	55.43	2.25	3.71
1	13	42.8	1.8	2.2
1	Total Avg	66.7	2.08	6.01
2	1	182.6	2.58	5.4
2	2	252.67	3.24	4.33
2	3	134.5	1.82	3.67
2	4	178	2.6	3.67
2	5	132.67	2.11	3.33
2	6	137.83	2.02	3.67
2	7	151.5	2.44	5
2	8	163.67	2.2	4.33
2	9	109	1.37	3.6
2	10	150.83	2.5	3.67
2	11	129.5	2.46	5
2	12	102.29	1.89	3.29
2	13			
2	Total Avg	147.52	2.22	3.98
3	1	169.4	3.23	5
3	2	137	2.6	3
3	3	81.5	1.92	3
3	4	81.5	1.98	2.17
3	5	72	1.5	2.5
3	6	88.25	2.08	3
3	7	78	1.7	2.25
3	8	84.4	1.73	2.83
3	9	44.2	0.75	2.4
3	10	108	2.26	2.25
3	11	104	2.58	3.25
3	12	76	1.96	3.17
3	13	57.2	1.66	1.6
3	Total Avg	88.35	1.96	2.8
4	1	134.8	3.14	4.2
4	2	137	4.44	3.33
4	3	163.33	3.69	3.67
4	4	184.67	4.03	4.5
4	5	167.67	3.41	3.83
4	6	163	3.63	3.67
4	7	182.25	3.81	4
4	8	198.17	3.93	4.5
4	9	131	2.91	4.4
4	10	176.5	3.74	3.5
4	11	160.5	3.71	3.25
4	12	125.83	2.98	3.57
4	13	185.8	4.36	4.2
4	Total Avg	163.46	3.65	3.91
5	1	222.8	3.26	4.8
5	2	205.67	3.37	4.33
5	3	125.83	1.92	3.33
5	4	161.17	2.41	4
5	5	142.17	2.01	3.67
5	6	162	2.17	4.83
5	7	193	2.74	5
5	8	185.17	2.34	4.83
5	9	133	1.71	4.6
5	10	151.33	2.35	4
5	11	176	2.82	5.25
5	12	143.14	2.34	5.14
5	13	183	3.08	4.8
5	Total Avg	164.57	2.44	4.48
6	1	162.4	2.72	3.6
6	2	138.33	2.79	2
6	3	72.5	1.74	2
6	4	83.33	1.84	1.83
6	5	76.4	1.52	1.67
6	6	109.75	1.71	2.8
6	7	54.33	1.21	2.33
6	8	87.33	1.44	2.5
6	9	73	1.3	2
6	10	73.5	1.7	2.67
6	11	70.5	1.69	2.5
6	12	73.14	1.71	2.71
6	13	36.5	1.76	1.8
6	Total Avg	84.62	1.76	2.34
7	1	174.6	3.17	5
7	2	162.33	3.45	4.67
7	3	144	3.14	4
7	4	134.33	3.31	4.5
7	5	153.17	4.13	4
7	6	128.67	2.89	4.33
7	7	142	2.77	4.25
7	8	139	2.47	3.83
7	9	163	3.15	5
7	10	Time-Loss Injury	Time-Loss Injury	Time-Loss Injury
7	11	Time-Loss Injury	Time-Loss Injury	Time-Loss Injury
7	12	Time-Loss Injury	Time-Loss Injury	Time-Loss Injury
7	13	Time-Loss Injury	Time-Loss Injury	Time-Loss Injury
7	Total Avg	146.93	3.16	4.33
8	1	137.6	2.74	3.4
8	2	136	2.43	2.33
8	3	79.33	1.81	2.83
8	4	153.5	3.56	4.67
8	5	127.5	3.02	3.5
8	6	150	3.42	4.17
8	7	166.5	3.71	5.25
8	8	160.83	3.49	5.67
8	9	108.8	2.5	4
8	10	134.5	3.63	3.75
8	11	141.5	3.58	4.25
8	12	75.75	1.94	3
8	13	27.5	1.89	2
8	Total Avg	127.79	2.96	3.9
9	1	183.8	2.83	5
9	2	154.33	2.64	4
9	3	110.83	1.82	2.67
9	4	152.33	2.6	4
9	5	140	2.21	3.83
9	6	153.5	2.1	4.67
9	7	178.75	2.32	5
9	8	212	2.95	5.17
9	9	154	2	5.2
9	10	154.83	2.83	3.83
9	11	138.75	2.92	4
9	12	135	2.51	4.29
9	13	208.4	4.13	4.6
9	Total Avg	158.7	2.58	4.3
10	1	215	3.44	5.8
10	2	208.33	3.18	4.33
10	3	144	2.6	4.83
10	4	118.83	2.3	3.5
10	5	84.4	2.03	4.33
10	6	124.17	2.19	4.67
10	7	136.5	2.53	5.25
10	8	176.8	2.96	5.8
10	9	113	2.36	5
10	10	112.67	2.47	4.5
10	11	120.5	2.83	4.25
10	12	86.71	1.94	3.71
10	13	77	3.15	5
10	Total Avg	128.97	2.55	4.64
11	1	143.4	3.5	5
11	2	164.33	4	4
11	3	127.17	3.34	4.33
11	4	125.67	3.54	4.17
11	5	111.67	2.94	3.83
11	6	111	3.06	4
11	7	132	3.83	4.5
11	8	118	3.85	4.5
11	9	102.2	2.87	4.4
11	10	99.5	3.58	3.5
11	11	78.75	3.64	4
11	12	82.67	2.89	3.71
11	13	111.6	4.23	4.8
11	Total Avg	114.35	3.44	4.19
12	1	112	3.38	5.4
12	2	96.67	2.84	2.67
12	3	61.17	1.48	3.2
12	4	71.5	2.08	3.4
12	5	54.2	1.69	3.67
12	6	51	1.69	3
12	7	51.75	1.8	3.75
12	8	55.17	1.58	3.83
12	9	45.4	1.3	3.4
12	10	46.67	1.61	2.5
12	11	46.5	1.85	2.75
12	12	45.43	1.94	2.71
12	13	33	2.8	3
12	Total Avg	59.45	1.91	3.35
13	1	129	3.25	5.6
13	2	169.33	3.27	4.67
13	3	76.5	3.26	3.67
13	4	103.17	3.62	3.67
13	5	109.17	3.03	3.67
13	6	113.83	3.38	4.17
13	7	140.75	3.75	5
13	8	137.17	3.72	4.67
13	9	82.6	2.34	4
13	10	112.67	3.45	3.5
13	11	97	2.9	3.67
13	12	71.14	2.43	3.86
13	13	126.6	4.12	4.6
13	Total Avg	109.61	3.26	4.16
14	1	163.6	3.64	5
14	2	144.33	3.37	4.67
14	3	33.67	0.7	2.33
14	4	144	1.64	2.33
14	5	64.8	1.6	2
14	6	63	1.74	2.83
14	7	143.33	3.38	2.25
14	8	58	1.7	2.83
14	9	39.4	0.86	2.8
14	10	57.33	1.38	2.67
14	11	61	1.74	3.25
14	12	79.33	2.18	3.5
14	13	53.33	2.86	2.33
14	Total Avg	82.72	1.96	2.97
15	1	157.4	2.81	5.6
15	2	188.33	3.29	5
15	3	130.17	2.67	5.83
15	4	135.4	2.7	5.2
15	5	77.2	1.66	4.33
15	6	126.33	2.42	5
15	7	155	2.7	5.25
15	8	138.5	2.53	5
15	9	107	2.14	6
15	10	99.83	2.41	3.5
15	11	113.25	2.49	4.5
15	12	84.43	2.21	3.71
15	13	118	2.55	5
15	Total Avg	121.99	2.48	4.87
17	1	183.6	2.45	4.4
17	2	188	2.75	4.33
17	3	134	2.32	3.83
17	4	117.67	2.42	3.6
17	5	135	2.36	3.83
17	6	140.17	2.34	4
17	7	122.75	2.34	3.75
17	8	154.5	2.51	4.83
17	9	119.6	1.88	5
17	10	130.67	2.32	3.17
17	11	123.75	2.49	3.5
17	12	111.43	1.88	3.43
17	13	141.4	2.15	4.2
17	Total Avg	136.62	2.3	3.97
18	1	176	3.63	5.8
18	2	185.67	3.93	4.67
18	3	135.33	3.11	4.67
18	4	114.5	2.65	4
18	5	105.67	2.43	3.83
18	6	117.17	2.65	4.67
18	7	132.5	2.89	4.5
18	8	97.6	2.67	4.67
18	9	70.6	1.69	3.2
18	10	101.17	2.09	4.5
18	11	91	2.37	4
18	12	92.86	2.32	4
18	13	78	4.2	3
18	Total Avg	112.57	2.76	4.28
19	1	233.6	3.85	5.6
19	2	181.33	4.7	2.33
19	3	182	3.49	4.17
19	4	239.4	4.53	4.2
19	5	182.67	3.44	3.83
19	6	152	2.81	3.2
19	7	188	3.28	4.33
19	8	232.5	3.8	4.17
19	9	170	3.14	4.4
19	10	210.17	4.1	3
19	11	142.5	3.45	3
19	12	138.14	3.04	3.83
19	13	191.4	1.92	3.8
19	Total Avg	188.19	3.47	3.88
20	1	233.6	2.49	4.6
20	2	177.33	2.08	4.33
20	3	99.33	1.56	1.83
20	4	129	1.6	2
20	5	106.33	1.18	2.33
20	6	123.5	1.52	2.5
20	7	156	1.62	3.5
20	8	140.5	1.45	2.5
20	9	93.4	1.3	4.4
20	10	116.67	1.5	2.5
20	11	128.75	1.88	2.5
20	12	91.83	1.44	2.29
20	13	101.4	1.58	2
20	Total Avg	127.29	1.6	2.75
21	1	147.2	3.12	5.4
21	2	123	2.34	4
21	3	60.83	1.59	2.5
21	4	42.33	1.66	2.17
21	5	59.17	1.31	2.67
21	6	72.5	1.77	2.6
21	7	50.25	1.29	2
21	8	61	1.35	2.2
21	9	20.2	0.43	2.4
21	10	65.75	1.5	2.5
21	11	68	1.7	3
21	12	36.17	2.04	3.33
21	13	29	2.27	2.2
21	Total Avg	61.48	1.71	2.81
22	1	131.8	3.58	5.2
22	2	136	4.07	2.33
22	3	95	3.01	4.33
22	4	60.67	2.32	2.83
22	5	52	1.69	2.67
22	6	83	2.45	4.83
22	7	62.5	2.04	3.5
22	8	48	1.67	3.67
22	9	48	1.59	3.4
22	10	48.17	1.78	3.33
22	11	63.25	2.1	4
22	12	72.14	2.51	4.14
22	13	54	2.39	2.8
22	Total Avg	71.38	2.35	3.67
23	1	223	3.69	6.4
23	2	77.33	1.74	1
23	3	170.5	3.27	4.17
23	4	159.5	2.49	4.17
23	5	125.2	2.03	3.6
23	6	169.33	2.88	4.83
23	7	123.25	2.13	4.25
23	8	175.33	3.11	4.83
23	9	115.6	2.52	5
23	10	186.4	3.57	3.83
23	11	166	3.71	4.5
23	12	120.17	2.55	4.83
23	13	194.6	4.32	5.6
23	Total Avg	157.3	2.96	4.6
24	1	254.2	4.33	5.4
24	2	269.67	5.19	3.67
24	3	197.67	4.4	4
24	4	206.67	4.54	4
24	5	140.8	3.1	4.17
24	6	193.83	4.03	3.67
24	7	217.25	4.32	5
24	8	233.83	4.58	4.5
24	9	167.6	3.28	4.4
24	10	187.17	4.2	3.67
24	11	173.75	4.16	4
24	12	147.29	3.11	3.71
24	13	201.8	4.51	5
24	Total Avg	196.19	4.09	4.22
25	1	153.2	3.08	5.8
25	2	159.33	3.41	4.67
25	3	126	3.14	4.67
25	4	111.67	2.76	4
25	5	104.83	2.37	4.17
25	6	104.5	2.48	4.5
25	7	96	2.27	4.75
25	8	88.5	1.97	4.67
25	9	32.2	0.83	2.6
25	10	88	2.42	3.83
25	11	95.25	2.7	3.5
25	12	107.29	2.62	4.29
25	13	151.2	4.28	4.8
25	Total Avg	107.51	2.61	4.32
Team Average	1	179.15	3.23	5.11
Team Average	2	162.86	3.28	6.29
Team Average	3	114.65	2.52	3.71
Team Average	4	127.12	2.71	3.54
Team Average	5	107.73	2.28	3.39
Team Average	6	122.64	2.5	3.93
Team Average	7	130.19	2.62	4.09
Team Average	8	133.96	2.59	4.15
Team Average	9	93.54	1.89	3.97
Team Average	10	116.44	2.6	3.4
Team Average	11	108.63	2.68	3.71
Team Average	12	94.28	2.3	3.66
Team Average	13	117	3.06	3.7
Team Average	Total Avg	122.05	2.6	3.96
